# Evaluation of two immunodiagnostic tests for early rapid diagnosis of leptospirosis in Sri Lanka: a preliminary study

**DOI:** 10.1186/s12879-015-1080-z

**Published:** 2015-08-11

**Authors:** Egwin J. Eugene, Shiroma M. Handunnetti, Shalini A. Wickramasinghe, Thilini L. Kalugalage, Chathuraka Rodrigo, Hasith Wickremesinghe, Nandana Dikmadugoda, Pranitha Somaratne, H Janaka De Silva, Senaka Rajapakse

**Affiliations:** Institute of Biochemistry, Molecular Biology and Biotechnology, University of Colombo, Colombo 03, Sri Lanka; Department of Clinical Medicine, Faculty of Medicine, University of Colombo, Colombo 08, Sri Lanka; Base Hospital, Homagama, Colombo, Sri Lanka; Department of Bacteriology, Medical Research Institute, Colombo 08, Sri Lanka; Department of Medicine, Faculty of Medicine, University of Kelaniya, Ragama, Sri Lanka

## Abstract

**Background:**

Leptospirosis is often treated based on clinical diagnosis. There is a need for rapid laboratory diagnosis for this condition. The aim of this study was to compare the diagnostic accuracy of two rapid IgM based immunodiagnostic assays with the microscopic agglutination test (MAT), in acute leptospirosis infection.

**Methods:**

MAT, IgM based immunochromatographic test (Leptocheck-WB) and IgM ELISA were performed using acute sera of patients clinically suspected to have leptospirosis (*n* = 83). Bayesian latent class modeling was used to compare the accuracy of these tests.

**Results:**

Percentage positivity for MAT, Leptocheck-WB, and IgM ELISA were 48.1, 55.3, and 45.7 % respectively. Bayesian latent class modeling showed a combined positivity rate of leptospirosis of 44.7 %. The sensitivity of MAT, Leptocheck-WB and IgM ELISA were 91.4, 95 and 81.1 %, and specificity were 86.7, 76.4 and 83.1 %, respectively.

**Conclusions:**

Leptocheck-WB has high sensitivity, and, because it is quick and easy to perform, would be a good screening test for acute leptospirosis infection. IgM ELISA has good specificity, and is comparable with MAT; given that it is easier to perform and more widely available than MAT, it would be a more appropriate confirmatory test for use in hospitals with limited access to a specialized laboratory.

## Background

Leptospirosis is a zoonosis of ubiquitous distribution, caused by infection with pathogenic *Leptospira* species [1]. In Sri Lanka, disease notification data shows a steady increase in the incidence of leptospirosis over the last two decades, which is attributable to disease emergence as well as improved surveillance [2]. During a large outbreak which occurred in 2008, the reported incidence rate was 7099 cases (35.7 per 100,000 population), with 204 deaths. The disease continues to affect large numbers of people each year, especially those in the farming community, resulting in significant morbidity and economic impact. In 2013, there were 4308 reported cases, with 78 deaths, giving an epidemiological prevalence of 21.5 per 100,000 population.

Clinical features of leptospirosis are similar to dengue and many other tropical infectious diseases in Sri Lanka; thus early laboratory confirmation of the diagnosis will help guide clinicians to institute appropriate treatment early in the course of the illness and plan appropriate resource allocation, potentially preventing complications and death. In most countries, accurate laboratory diagnosis is a challenge. Microscopic agglutination test (MAT) is generally considered the standard immunological test used for diagnosis of leptospirosis. Laboratory confirmation based on MAT is a much delayed process, and it is technically challenging, requiring experience laboratory scientists to perform the tests, and the maintenance of live leptospira cultures. Often, treatment is initiated on the basis of clinical assessment with subsequent laboratory confirmation using MAT. There is a need for rapid diagnostic tests which can yield accurate results early on in the course of the clinical illness. Clinical leptospirosis is a biphasic illness, with a leptospiraemic phase occurring from the 4^th^ to 7^th^ day followed by a leptospiruric phase that can last for 4-30 days. Leptospiruria coincides with the immune phase which begins with the appearance of IgM antibodies [3, 4]. Therefore, IgM based serology is of diagnostic value during this phase. Many rapid serodiagnostic assays are available, such as IgM based microplate enzyme-linked immunosorbant assay (ELISA), IgM dot ELISA test, IgM dipstick, latex agglutination test and haemagglutination assay [5–8]. These rapid tests are easy to perform and read, although their scientific validity with respect to sensitivity and specificity need further evaluation. To date, only one study has been reported from Sri Lanka evaluating commercially available rapid immunodiagnostic kits [9]. This study evaluated the microplate ELISA (Institut Viron Serion GmbH, Germany) and showed very low sensitivity and specificity values. Other rapid serological diagnostic assays have not been evaluated against a reference test in Sri Lanka.

This study compared the efficacy of two rapid immunodiagnostic assays for the detection of leptospira IgM antibodies, *i.e*., IgM based immunochromatography test (Leptocheck-WB test) and IgM based microplate ELISA, together with MAT. MAT detects both IgM and IgG antibodies. The validity of MAT as a gold standard could be considered imperfect, as MAT positivity may result from previous infection, while a MAT-negative result in the presence of positivity by an IgM-specific assay may occur due to either low sensitivity or serovar specificity of MAT. In settings such as this, Bayesian latent class modeling has been suggested to be a more suitable method for evaluating diagnostic tests, as it assumes that all tests are imperfect [7, 10]. We therefore compared these tests using Bayesian latent class modeling.

## Methods

### Patients

Serum samples were collected from patients clinically suspected to have acute leptospirosis, admitted to the National Hospital, Colombo, (NHSL) and Base Hospital, Homagama (BHH), Sri Lanka during the period June to September 2010. The following criteria, based on World Health Organisation-Leptospirosis Epidemiology Research Group (WHO-LERG) Epidemiological criteria were used to define a suspected case of leptospirosis; acute febrile illness with any of the following: headache, myalgia, arthralgia, conjunctival suffusion, meningeal irritation, anuria, oliguria, proteinuria, jaundice, hemorrhages, cardiac arrhythmia, skin rash; or with a contact history of exposure to water or soil contaminated with urine of infected animals. Patients with a clear alternative diagnosis were excluded. MAT, IgM Leptocheck-WB test, and ELISA were performed on acute serum samples from these patients. Informed written consent was obtained from all patients included in the study. There were 83 patients presenting with clinically suspected acute leptospirosis; mean age 39.9 years (SD ±15.2), male: female ratio was 20:1.

### Laboratory methods

The Leptocheck-WB test kit was obtained from Zephyr Biomedicals, India, and the IgM ELISA Leptospira kit from Diagnostic Automation Inc., USA. MAT was performed at the Department of Bacteriology, Medical Research Institute, Sri Lanka. For MAT, sera which gave an agglutination of at least 50 % of the leptospires (compared with the control antigen) was considered to be positive, with a serum titre of ≥400 considered the positive threshold [11]. *Leptospira biflexa* serovar Patoc strain Patoc-1, which is an indicator strain, was used as antigen in all three tests, *i.e*., MAT, Leptocheck-WB and IgM ELISA.

The Leptocheck-WB test kit components were used according to manufacturer’s instructions. [13] Briefly, contents were placed at room temperature (RT; 25 °C), and the test device was labeled with the patient's identity (patient’s code). Serum sample (10 μl) was added into the sample port twice and 5 drops of sample running buffer was dispensed into the buffer port immediately. The device was kept at RT and the results were read at the end of 15 min. Depending on various intensities observed, they were subjectively scored as 0, 1+, 2+, 3+ or 4+ based on the intensity of colour of the antigen band, using a colour reference diagram. A result of 1+ or greater was considered positive according to the manufacturers’ instructions.

The antigen coated on the IgM ELISA kit used was whole cell lysate from *Leptospira biflexa* patoc-1. The IgM-ELISA kit was used according to manufacturer’s instructions [10]. Briefly, 1:40 dilutions of serum samples were prepared using the dilution buffer provided with the ELISA kit. Rheumatoid factor (RF) absorbent (40 μl) was added to 100 μl of diluted test serum (patient sera and healthy control sera), mixed well, incubated in the tubes for 5 min and then added to ELISA plate. Pre-diluted negative and positive controls provided were added to the ELISA plate, and the RF absorbent added according to manufacturers’ instructions. The plate was incubated at RT for 10 min. The contents were then washed 3 times with the diluted wash buffer provided, and two drops of enzyme conjugate were added to each well and incubated at RT for 10 min. The plate was washed 3 times with wash buffer and two drops of chromogen was added to each well and incubated at RT for 5 min. The reaction was stopped by adding 2 drops of the stop solution per well, mixed well, and the plate was read at 450 nm using an ELISA plate reader (ELx800- universal microplate reader, Bio-Tek, Instruments INC, Canada.). The cut-off value was ≥ 0.848, calculated by plotting the Receiver Operating Characteristic (ROC) curve.

### Statistical analysis

Data was entered and analyzed using SPSS® statistical software version 17. Descriptive analysis was done using percentages. The MICE tool (Modelling for Infectious Diseases Centre, Mahidol-Oxford Research Unit, Thailand [http://mice.tropmedres.ac/home.aspx]) was used for Bayesian latent class modeling. The use of Bayesian latent class models to determine the accuracy of diagnostic tests where the gold standard is imperfect has been described elsewhere [7].

### Ethics statement

Ethics approval (Ref No. EC/09/054) was obtained from the Ethics Review Committee of the Faculty of Medicine, University of Colombo.

## Results

For the 83 patients included in the study, the positivity obtained by the three tests, *i.e*., MAT, immunochromatography and IgM ELISA were 48.1, 55.3, and 45.7 % respectively. Positivity by all three tests was 32.5 %. The combined positivity for Leptocheck-WB and MAT was 41.0 % whereas positivity with Leptocheck-WB and IgM ELISA was 37.3 % and IgM ELISA and MAT was 34.9 % (Table 1).

**Table 1:** Detection of anti-leptospira antibodies in human sera, by MAT, Leptocheck-WB and IgM ELISA assays

Number of samples by test (%)	MAT	Leptocheck-WB	IgM ELISA
27 (32.5)	+	+	+
7 (8.4)	+	+	-
2 (2.4)	+	-	+
4 (4.8)	+	-	-
4 (4.8)	-	+	+
8 (9.6)	-	+	-
6 (7.2)	-	-	+
26 (31.3)	-	-	-
Total 83 (100.0)			
Positivity by single test	48.1 %	55.4 %	46.9 %

Based on Bayesian latent class modeling, the combined positivity rate (*i.e*., percentage positive based on any of the tests) of leptospirosis was 44.7 % (95 % Credible Interval [CrI] 30.8-60.8). The sensitivity of MAT, Leptocheck-WB and IgM ELISA were 91.4, 95 and 81.1 %, and specificity were 86.7, 76.4 and 83.1 %, respectively. Leptocheck-WB had the highest sensitivity, with a negative predictive value of 95.1 %. MAT still had the highest specificity, although the specificity of IgM ELISA was comparable (positive predictive values 84.7 and 79.6 %) (Table 2). Good convergence was also seen for all parameters by using the MICE tool (see methods). Bayesian p-values for the profiles ranged from 0.418-0.630, indicating good fitness.

**Table 2:** Comparison of diagnostic accuracy of MAT, Leptocheck-WB, and IgM ELISA, using Bayesian latent class modeling

Parameters	Bayesian latent class model %(95 % credible interval)
Combined positivity rate	44.7 (30.8 - 60.8)
MAT	
Sensitivity	91.4 (72.2 - 100)
Specificity	86.7 (70.7 - 99.3)
PPV	84.7 (63.6 - 99.3)
NPV	92.7 (71.2 - 100)
Leptocheck-WB	
Sensitivity	95.0 (79.3 - 100)
Specificity	76.4 (60.8 - 93.2)
PPV	76.5 (56.2 - 94.3)
NPV	95.1 (75.9 - 100)
IgM ELISA	
Sensitivity	81.1 (62.4 - 97.3)
Specificity	83.1 (68.8 - 94.8)
PPV	79.6 (60.4 - 94.4)
NPV	84.5 (64.0 - 98.2)

*PPV* positive predictive value, *NPV* negative predictive value

## Discussion

The primary objective of this preliminary study was to compare the relative accuracy and suitability of two rapid immunodiagnostic tests, *i.e*., an IgM based immuno-chromatographic test, *i.e.*, Leptocheck-WB, and a microplate IgM ELISA, together with the conventional MAT test which is used most widely for diagnosis currently.

Leptocheck-WB is easy to perform, is a rapid method which takes only 15 min, and requires only a single dilution with no requirement of special equipment. In contrast, IgM ELISA has several steps in its procedure with a time duration of about 50-60 min and requires an ELISA plate reader. Leptocheck-WB test gave consistent results and the bands were stable for more than 12 months. Both tests were relatively inexpensive with a cost of less than 200 SLR (1.6 USD/test). The positivity produced by Leptocheck-WB test was 55.3 % while that for IgM ELISA was 45.7 %. We suggest, based on our findings, that Leptocheck-WB would be an appropriate screening test for leptospirosis, especially in hospitals with limited laboratory facilities. Leptocheck-WB can easily be performed as an individual test, either with serum or whole blood samples, the assay contents are stable and could be transported and stored at ambient temperatures. The test is a portable package, and is easy to interpret, with no requirement for skills in handling specialized equipment such as an ELISA microplate reader. Previous studies have shown variable sensitivity and specificity of Leptocheck-WB. One study in South Gujarat showed Leptocheck-WB to have relatively low sensitivity (78.7) and higher specificity (88.3 %) [12], compared to the sensitivity and specificity of 95 and 76.5 % in our study. In contrast, another study, also in South Gujarat, showed Leptocheck-WB to have very high sensitivity (98.4 %), while specificity was similar to the previous study (86.9 %) [11].

Despite the high specificity and sensitivity shown with MAT, this test is more difficult to perform, requires a specialized laboratory and trained staff, and is thus not available routinely to clinicians in remote rural hospitals. Current WHO guidelines recommend a MAT titer of ≥400 in a single or paired serum sample or a 4-fold increase in MAT in acute and convalescent sera for laboratory conformation of Leptospirosis [13]. In our study, a MAT titer of ≥400 in acute serum was considered as the reference standard. However, MAT measures both IgG and IgM; the duration for which IgG and IgM levels persist after acute infection is not clearly known. Thus, MAT positivity could reflect previous rather than acute infection. Our own data suggests that IgM levels decrease rapidly 3 months after acute infection (unpublished data). We suggest that IgM ELISA would be a more suitable test than MAT for use by clinicians treating patients with acute infections, for the following reasons. Firstly, the fact that MAT is only available in larger laboratories leads to delays and difficulties in obtaining early results, while IgM ELISA is more likely to be available at the point of care. Secondly, there is mounting evidence that IgM ELISA maybe more sensitive in identifying infection early on in the course of illness, which will provide a definite advantage to the treating clinician to prioritize healthcare resources in a timely manner. A meta-analysis of studies of ELISA in leptospirosis showed that the pooled sensitivity for IgM ELISA tests was 80.4 (79.2–81.5), and specificity was 94.4 % (93.9–94.9) [14], values which are similar to our study. Nonetheless, there is considerable heterogeneity seen in ELISA tests for leptospirosis, in previous studies [14]. The fact that the commercial ELISA method used in our study has good sensitivity and specificity is of importance in the local context.

Arguably, the main limitation of these three tests is the fact that they are genus specific for *Leptospira biflexa.* There is considerable interest in developing serovar specific immunological tests, and also in-house immunodiagnostics which would give higher diagnostic specificity and sensitivity for local serovars. Nonetheless, our results show reasonably high accuracy with these commercial tests, making these pragmatic alternatives to guide clinicians treating patients with leptospirosis in resource limited developing country settings.

Interestingly, pooled meta-analysis shows that, based on ELISA, IgM antibodies are more specific that IgG antibodies for leptospirosis [14]. It is postulated that this could be due other febrile illnesses causing a non-specific rise in IgG, resulting in false positives. If this were the case, it would also suggest that MAT, which tests both IgG and IgM antibodies, is less specific than IgM. Nonetheless, MAT is likely to remain the epidemiological gold standard for diagnostic confirmation of leptospirosis infection, eventually to be replaced by genomic techniques.

This was a preliminary study with limited sample size, and our findings emphasize the need for a larger study with greater statistical power. Such a study is currently in progress by our research team. This study was limited to one season and emphasizes the need for coverage of different geographical regions of the country, a longer study period to include seasonal variations and a larger sample size. Factors that could affect the reported sensitivity and specificity of Leptocheck-WB test may include its genus specific nature and inability to react and recognize the infecting serovar specific IgM antibodies.

## Conclusions

Our preliminary findings suggest that Leptocheck-WB would be a suitable screening test, and IgM ELISA an appropriate confirmatory test for patients with acute leptospirosis. MAT will remain the reference standard for epidemiological purposes, especially if PCR is not available, however it’s availability is limited.

## Abbreviations

MAT: Microscopic agglutination test
IgM: Immunoglobulin M
IgG: Immunoglobulin G
PCR: Polymerase chain reaction
ELISA: Enzyme-linked immunosorbant assay
RT: Room temperature
MICE: Modeling for infectious diseases centre
CrI: Credible interval
